# Weakly Supervised Learning for Categorization of Medical Inquiries for Customer Service Effectiveness

**DOI:** 10.3389/frma.2021.683400

**Published:** 2021-08-02

**Authors:** Shikha Singhal, Bharat Hegde, Prathamesh Karmalkar, Justna Muhith, Harsha Gurulingappa

**Affiliations:** ^1^ Group Data Science, Merck Data Office, Merck Life Sciences, Bengaluru, India; ^2^ Faculty of Electrical Engineering and Information Technology, Darmstadt University of Applied Sciences, Darmstadt, Germany; ^3^ Global Medical Information, Merck Healthcare KGaA, Darmstadt, Germany; ^4^ Group Data Science, Merck Data Office, Merck KGaA, Darmstadt, Germany

**Keywords:** natural language processing, medical information, deep learning, weakly supervised learning, customer inquiry

## Abstract

With the growing unstructured data in healthcare and pharmaceutical, there has been a drastic adoption of natural language processing for generating actionable insights from text data sources. One of the key areas of our exploration is the Medical Information function within our organization. We receive a significant amount of medical information inquires in the form of unstructured text. An enterprise-level solution must deal with medical information interactions via multiple communication channels which are always nuanced with a variety of keywords and emotions that are unique to the pharmaceutical industry. There is a strong need for an effective solution to leverage the contextual knowledge of the medical information business along with digital tenants of natural language processing (NLP) and machine learning to build an automated and scalable process that generates real-time insights on conversation categories. The traditional supervised learning methods rely on a huge set of manually labeled training data and this dataset is difficult to attain due to high labeling costs. Thus, the solution is incomplete without its ability to self-learn and improve. This necessitates techniques to automatically build relevant training data using a weakly supervised approach from textual inquiries across consumers, healthcare professionals, sales, and service providers. The solution has two fundamental layers of NLP and machine learning. The first layer leverages heuristics and knowledgebase to identify the potential categories and build an annotated training data. The second layer, based on machine learning and deep learning, utilizes the training data generated using the heuristic approach for identifying categories and sub-categories associated with verbatim. Here, we present a novel approach harnessing the power of weakly supervised learning combined with multi-class classification for improved categorization of medical information inquiries.

## Introduction

There is an exponential growth in unstructured data within Medical Information functions in pharmaceutical organizations. This ever-growing data remains untapped to identify actionable insights and recommendations from textual data that can generate significant patient value. Hence arises a strong business need to understand and analyze the customer inquiries which can drive strategic decisions related to product quality, labeling, safety, marketing, supply, and customer service effectiveness.

In recent years, machine learning (ML) and deep learning (DL) have created a real-world impact in various tasks, especially in supervised learning like classification and regression. Predictive modeling requires a huge amount of training examples for learning. These training examples have two main components. The first ones are the features that describe the event and others are labels that indicate the outcome. Most of the successful techniques require ground-truth labels to be given for a large training set. However, there is a limitation that these models are reliant on a huge set of manually labeled training data. These manually labeled training data are difficult to attain due to the high cost of the labeling process. It often requires experts for months to assemble the labeled data especially when domain knowledge is needed. Labeling training data is the largest bottleneck in generating the machine learning models. For these reasons, researchers are switching from strongly supervised approaches to weakly supervised ones. In the weakly supervised approach, the training data can be generated using heuristic rules, patterns, and external knowledgebase. Any machine learning or deep learning techniques explained in this article have been collectively named as *machine learning* alone.

As a part of our approach, we leveraged weakly supervised techniques on medical information inquiries captured and received by the Global Medical Information business. On a day-to-day basis, our organization receives customer inquiries, feedback, complaints, and issues through their contact centers. A large portion of this data is unstructured verbatim text. The business analytics team currently spends a significant amount of time manually extracting and mining each interaction transcript to identify the trend of topics. To avoid this manual effort, we leveraged a weakly supervised approach for tagging categories to each interaction of customer inquiry data. Our text analytics engine is coupled with contextual NLP/ML and domain-specific knowledgebase to generate a comprehensive, robust, scalable and near real-time solution for automatically categorizing medical contact center verbatim and deriving actionable insights.

### Literature Survey

A detailed literature survey was done to understand how pharmaceutical companies are leveraging NLP and ML to analyze the customer inquiry data and generate insights out of them. Due to the sensitivity and confidentiality of Medical Information inquiries data as well as the lack of availability of public benchmark datasets, published research in this domain is very limited. We reviewed literature related to the research around NLP approaches applied to extract medical information from text (Torii et al., 2011). performed concept extraction to identify phrases referring to concepts of interest in unstructured text. The investigation was done to check the performance of machine learning taggers for clinical concept extraction, particularly the portability of taggers across documents from multiple data sources. Research had been carried out to categorize the clinical unstructured data. For example (Szlosek and Ferrett, 2016), used machine learning classification algorithms like decision tree, SVMs, and k-nearest neighbor along with natural language processing algorithms to accurately evaluate a clinical decision support rule through an electronic medical records system and compared it against manual evaluation. It provides a view into the current state of methodologies proven effective for the application of NLP in the pharmaceutical domain. In recent years, ML has created an impact in supervised learning like text classification. Significant work has been done on weakly supervised learning to remove the dependencies on labeled training data (Zhou, 2018). explained the issues faced in supervised learning and how weakly supervised learning can be used to overcome those problems. The article shows research progress in weakly supervised learning and different types of weak supervisions like incomplete supervision, inexact supervision, and inaccurate supervision (Jain et al., 2016). shows how biomedical information can be extracted from curated data using a weakly supervised learning approach and describes a general approach on how biomedical information can be extracted from curated data using weakly supervised learning. The authors suggested to device a formula that represents problem statement as cost-sensitive learning from unclean labels. Furthermore, the authors recommended calculating the cost by a committee of weakly supervised classifiers which consider both cleaned data and original text (Kanavati et al., 2020). used various transfer learning and weakly supervised learning approaches to predict carcinoma in Whole Slide Images (WSIs) using Convolution Neural Network (CNN) based on the EfficientNet-B3 architecture. It provides a brief view into the current state of methodologies proven effective for the application of weakly supervised learning approaches in the healthcare domain.

## Business Challenges

Some of the challenges and drawbacks in the existing system are as follows:

a) Experts manually analyze interaction transcripts by random spotting and keyword searching for information. This process is time-consuming, expensive, labor-intensive, and prone to information overlook since only a small portion of data can be analyzed.
b) Due to manual analysis, business experts were unable to tap deep into inquiries due to a lack of analytics derived from the unstructured data. This led to losing some of the critical recommendations and actionable insights.

Additionally, the Medical Information business supports multiple channels across the world, and this adds to the complexity of data. Inquiries are generated in multiple languages across different continents. The data source contains data in more than twenty non-English languages. Also, a significant amount of noise gets added to the data due to the transcription of inquiries from channels like phone and web chats. The database contains approximately 180K inquiries from different types of sources over the last three years. The system captures additional information such as case type, contact class, product name, responses, letters, attachments, follow-ups, inquirer details, country of origin, and many more. It has a data privacy protection layer and helps in providing accurate information submitted by physicians, patients, sales representatives, scientists, nurses, and pharmacies.

## Solution Overview

The solution contains NLP/ML model which was systematically trained using weakly supervised learning for categorization of inquiries as well as a production-ready system where the model is deployed for near real-time categorization of inbound inquiries. However, this article focuses on the methodology applied for the development and evaluation of NLP/ML models.

**FIGURE 1:**
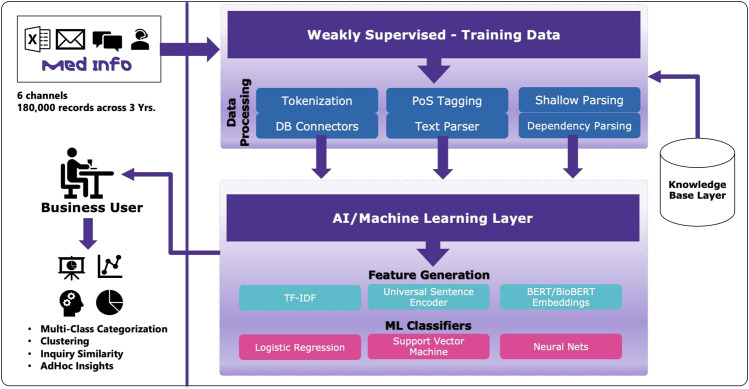
Overview of solution architecture.

As depicted in Figure 1 of the solution architecture, the solution has two major components. Due to multilingual data, all the non-English inquiries were translated into English using a commercial auto-translate engine. The first component comprises text preprocessing techniques such as tokenization, parts-of-speech tagging, and dependency parsing to generate features which would then leverage heuristics and knowledgebase to generate labeled data that can be used for training and evaluation. Identifying a group of medical experts who can spend time creating manually labeled datasets is a general challenge for enterprises. Therefore, instead of experts manually labeling these inquires, we created heuristic rules and patterns using domain knowledge to programmatically generate training data. These rules were crafted in cooperation with experts to ensure that they fit the classification objective as well as generate meaningful outcomes for business adoption.

Once the labeled data is available, various feature extraction techniques were investigated such as term frequency-inverse document frequency (TF-IDF), and embeddings generated using universal sentence encoders and BERT (Devlin et al., 2018). These features were then used as input to train and evaluate classification algorithms. Since there were multiple labels assigned to a single inquiry, the one-versus-rest strategy was used to solve the multi-class classification problem to improve the classification results. Also, various classification algorithms such as logistic regression, support vector machines, and neural networks were examined to generate the performance metrics.

## Methods

Our proposed weakly supervised approach contains two main modules. The first module uses heuristics and knowledgebase in combination with expert-defined rules to automatically generate the labeled data which can be used for training and evaluation. The second module systematically uses weakly supervised framework to train and evaluate supervised classification algorithms. Both the modules are explained in detail in the subsections below. Prior to the application of heuristic or machine learning, non-English records in the data were converted into English using a commercial auto-translation engine.

### Heuristically Labeling the Data

**FIGURE 2:**
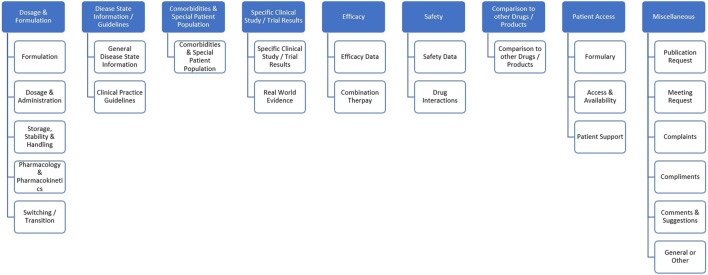
Categorization hierarchy used to label the inquiries. For the sake of algorithm development, only the sub-categories (i.e., level 2) were used.

Our approach is designed with the capabilities of ingesting data from the source system database into NoSQL database which is a part of our text analytics platform. The database contains approximately 180K inquiries. A sample of 60K inquiries received after January 01, 2019 were used as the working corpus. Figure 2 shows a two-level hierarchy used as a category tree which was employed for subsequent labeling. The hierarchy was developed semi-automatically by explorative data analysis as well as business knowledge from subject matter experts to fit the end-user needs.

Heuristic labeling identifies sub-categories conveying medical and non-medical context of the verbatim using expert crafted rules as well as an in-house knowledgebase. It uses a custom algorithm to identify the topics based on a chain of relations within input verbatim. The following steps show a set of techniques used for heuristic data labeling.

Step 1: Split the inquiries into sentences using an out-of-the-shelf sentence splitter. Since a significant portion of inquiries had mistyped sentences, spelling correction with symmetric delete spelling correction algorithm (SymSpell, 2020) was used to correct the misspelled words. Special character removal (non-ASCII characters, HTML tags, etc.) was applied to eliminate noise and an open-source python library was used to handle concatenated words within sentences (Compound Word Splitter, 2017). As a result of sentence reconstruction and handling concatenations, sentences generated for further processing were of higher quality than sentences resulting from out-of-the-shelf sentence splitter. Although a systematic evaluation was not employed to evaluate the quality of sentences, a spot check by data scientists and subject matter experts confirmed our observations.
Step 2: After splitting the inquiries into sentences, key phrases were identified using noun phrase chunker (SpaCy, 2015). An open-source dependency parser (SpaCy, 2015) was used to identify the chain of dependencies between different key phrases.
Step 3: Proprietary medical knowledgebase as well as expert curated lexicons were used to map the key phrases to entity types. Examples of entity types include treatments, medical conditions, diagnoses, pharmacological mechanism of action, and many more.
Step 4: A set of custom rules were devised to use key phrases, entity types, and dependency paths to automatically assign the labels (subcategories). A set of 450 rules were created across 23 labels. Rules were created based on standard definitions of labels shared in Supplementary Table 2. The example below shows how heuristic rules were applied to one of the inquiries in our dataset containing three sentences. Prioritization rules were applied when multiple labels can be associated with one sentence to ensure that only the most relevant label is assigned.
  Sentence-1: Would like to have any documentation available on allergies of Product-A.
  Key phrases: any documentation, allergies, Product-A.
  Entity mapping: any documentation (Document), allergies (Condition), Product-A (Treatment).
  Rule Applied: available <modifies> Document = Publication Request | Condition <modifies> Treatment AND Condition-Treatment pair occurs as adverse effect in knowledgebase = Safety data.
  Rule Prioritization: Publication Request overrules.
  Label: Publication Request.
  Sentence-2: Are there any cases of having allergies after taking Product-A?
  Key phrases: cases, allergies, Product-A.
  Entity mapping: allergies (Condition), Product-A (Treatment).
  Rule Applied: Condition <modifies> Treatment AND Condition-Treatment pair occurs as adverse effect in knowledgebase = Safety data.
  Label: Safety Data.
  Sentence-3: I want MSL to meet me and discuss this further.
  Key phrases: MSL.
  Entity mapping: MSL (Professional).
  Rule Applied: Professional <subject> meet = Meeting Request.
  Label: Meeting Request.
Step 5: As the last step, the known parent-child relationships between subcategories and root categories were utilized to assign root categories to sentences. However, the root-level categories were not used for machine learning. For example, if the subcategory label of a particular sentence is Formulation, or Dosage and Administration, then the sentence is mapped to Dosage and Formulation as a root category (refer to Figure 2). Additional information on the heuristic labeling process can be found in (Karmalkar et al., 2021). Definitions of subcategories and data distribution are shown in Supplementary Table 1.

As a result of heuristic labeling, out of 130K sentences, 110,614 sentences were tagged with a minimum of one subcategory. On the other hand, the remaining approximately 19K sentences were labeled as General or Other and they were not used for training or evaluation purposes. The subcategories will be referred to as labels in the remaining sections of this article.

### Multi-Class Classification

This section highlights the second part of our approach leveraging the concepts of ML classification for a multi-class data set. This section will provide a brief overview of feature extraction and two kinds of techniques investigated for classification. The section will also share some insights on potential challenges caused due to weak supervision and how they were addressed.

Results of heuristic labeling which contains sentences and programmatic labels were used to train and evaluate different classification models. The problem was treated as multi-class classification. Given a collection of sentences S = {S_1_,. . ., S_n_} and m target classes C = {C_1_,. . . C_m_} with *m* = 23, the multi-class text classifier aims to assign class label C_i_ ∈ C to each sentence S_i_ ∈ S. Existing traditional supervised text classification methods rely on large amounts of labeled data. In this work, we focus on developing the multi-class text classifier with weakly supervised technique where the labels were generated using heuristics. The entire working corpus was used for training and evaluation using the 10-fold cross-validation criteria. Due to the non-availability of a manually labeled ground truth dataset, a sample of 1,600 non-overlapping sentences i.e., leave-out validation set (can also be referred to as independent test set) was automatically labeled with the best performing trained model. Subsequently, the machine-generated labels over this leave-out validation set were manually evaluated by domain experts. Experts were asked to assess the quality of automatic ML-based labels as *correct*, *incorrect*, or *incomplete*. Results of the assessment were subsequently used to compute metrics which indicates the performance of the classification model. This was done to ensure that the model is not biased toward heuristic labeling process. Also, the results of cross-validation are not indicative of the performance of machine learning on a real-world problem since labels were heuristically assigned and they could be noisy. Cross-validation rather supported investigation of the right algorithm, features, and hyper-parameters that can be used to prepare a final robust model for real-world evaluation and application. Performance evaluated by experts on the leave-out validation set provides a good indication of the practical applicability of the weakly supervised learning approach. Sizes of training, leave-out validation, and real-world evaluation datasets are provided in Table 1.

**TABLE 1:** Sizes of training, leave-out validation, and real-world evaluation sets.

Description	Number of sentences
Training set	110,614
Leave-out validation set	1,600
Real world evaluation set	1978

### Feature Extraction

TF-IDF features (Qaiser and Ali, 2018) (Term Frequency - Inverse Document Frequency): Stop words from inquiries were removed and TF-IDF features were generated over n-grams with n ranging from one to five. Maximum features were limited to 20,000.

Universal Sentence Encoder (USE): After splitting inquiries into sentences, USE embeddings were generated for every sentence within inquiries. The universal Sentence Encoder (Cer et al., 2018) encodes the variable length inquiries into 512-dimensional vectors. The Universal-sentence-encoder-large/5 model was used to collect contextual embeddings. Stop words were not removed while creating the embeddings.

#### Classification With Logistic Regression and Linear SVMs

Experimentation was done using classical ML classifiers such as logistic regression and SVMs using a one-versus-rest strategy. These ML models use heuristically generated training data to train the classifier. While evaluating the classifier, we identified a data imbalance problem. In subsequent sections, we show the approach used to overcome the data imbalance issue.

### One-Versus-Rest Strategy

One of the common ways of solving multi-class classification is to build the multiple N binary classifiers each one trained independently from the other for a specific label. While training for the individual labels, instances belonging to that specific label are considered positive whereas the others are considered negative. This process repeats N times for a unique label in the training set.

When it was desired to classify a new sentence, the N-trained models were run, and the classifiers which output the positive labels with the highest probability were considered as subcategories. So, the approach uses logistic regression and SVMs with a one-versus-rest strategy (Rifkin and Klautau, 2004). Models were trained and evaluated using both n-gram TF-IDF and USE as features independently. During the experimentation, training was also performed using SVMs with different kernels. Due to the poor performance of non-linear kernels, investigations with SVMs were limited to the linear kernel.

### Data Imbalance

We observed significant imbalanced labels within the training data. During the initial phases of the experiment, classifiers were trained using a one-versus-rest approach using with TF-IDF features trained over the imbalanced training data. Models trained using the imbalanced data showed low recall and hence the overall low F1-score.

### Data Balancing Technique

To avoid the data imbalance issue, a penalty-based loss function (Kotsiantis et al., 2005) also called a cost-sensitive training approach was applied. This approach uses a penalized learning algorithm or a loss function that increases the cost of classification for minority classes. Therefore, minority class losses are updated faster than the majority class. Every class is then penalized by multiplying weight to the loss function so that weights are inversely proportional to class frequencies. At first, one-versus-rest strategy was applied for every label. Therefore, *N* 23) models were trained independently. For every label, the weight (Scikit-learn, 2020) was calculated as below:

$$Weight_{i}=\left(n\_samples\right)/\left(n\_classes\times bincount\left(y_{i}\right)\right)$$

*n_samples*= total number of samples

*n_classes* = total number of classes (*2* i.e., positive class and negative class).

*bincount* = number of training sample available for *i*th class $y_{i}$.

$y_{i}$ = *i*th class (*i* = {0,1} i.e., negative class or positive class based on one-versus-rest strategy).

For minority classes, the weight values were high whereas for the majority classes, weight values were low. This would avoid overtraining in some classes. This technique was applied for training all other models explained in the subsequent sections.

Logistic regression and linear SVMs were trained using TF-IDF features as well as USE embeddings. After the loss calculation, the weights were multiplied to penalize the loss. The performance of models was evaluated using the 10-fold cross-validation technique. When training the model with TF-IDF features, we investigated different values for maximum features and found that 20 K provides better results and these features were calculated at the sentence level. Whereas, for training with embeddings features, USE’s standard vectors of dimension 512 were used directly for model training and evaluation. Hyper-parameters used for training are provided in Table 2.

**TABLE 2:** Hyperparameters used for training the classifiers.

Logistic regression and TF-IDF features	Logistic regression and USE embeddings	Linear SVMs and TF-IDF features
Solver: lbfgs	Embeddings dimension: 512	Regularization parameter (C): 3
Regularize: L2 nJobs = -1	Solver: Lbfgs	Kernal: Linear
Class weight: Balanced	Regularize: L2 nJobs = -1	Class weight: Balanced
K_Fold = 5	Class_weight: Weight param as per training sample	
	K_Fold = 5	

#### Classification With Neural Network Architecture

In this section, we describe the approaches leveraging different transformer models (Vaswani et al., 2017) such as BERT, BioBERT (Lee et al., 2020), and XLNet (Yang et al., 2019) applied for multi-class classification. Most recently, the transformer models with pre-trained word embeddings that produce rich language knowledge representation can be used for fine-tuning the downstream supervised task with limited data. Even though the overall model size is large, the advantage of this approach is that models can be trained with a small amount of training data.

During investigations with logistic regression described in the previous section, the one-versus-rest strategy was used for the multi-class classification and train models as a set of binary classifiers. However, for the deep learning approach, the feed-forward neural network layer with 23 output neurons on top of the transformer encoder embedding layer was used as a classification layer. This paper (Cer et al., 2018) proposes that the embeddings at sentence level can be built using transformer-based architecture. This approach uses an encoding sub-graph of the transformer architecture. This kind of design uses attention to compute context-aware representations of words in a sentence that consider both the ordering and identity of all the other words. With minimal architectural modifications and less data, these transformer models can be used for multi-class classification (Venkatesan and Er, 2014).

### Sentence Representation

Sentences were represented as 768-dimension vectors by taking the pooled out of individual token vectors within sentences. For example, a sentence is represented as below:

[cls] She took acyclovir for her treatment as well [sep].

So, the sequence output will generate a vector of dimension [1, 10, 768] since there are 10 tokens including [CLS] and [SEP] and the pooled output will generate a vector of dimension [1, 1, 768] which is the embedding of [CLS] token. This output is then passed through a feed-forward neural network layer with an input layer of 768 and an output layer of 23.

### Loss Function

The output of the neural network layer was passed through the sigmoid layer. Since it was a multi-class classification problem, binary cross-entropy loss function [13] was used as shown below.

$$L=mean\left\{l_{1},l_{2},....l_{N}\right\}$$

$$l_{n}=-W_{n}\left[Y_{n}*\text{log}X_{n}+\left(1-Y_{n}\right)*log\left(1-X_{n}\right)\right]$$

$X_{n}$ = output of neural network.

$Y_{n}$ = target label.

$W_{n}$ = rescaling weight.

*N* = number of classes (i.e., 23).

L = mean loss.

$l_{i}$ = loss for label i.

### Handling Data Imbalance in Neural Network Architectures

As described in the earlier sections, the data imbalance issue was addressed by manually rescaling loss calculated for every batch of data before updating the model parameters. At first, we calculated the binary cross-entropy loss of all output neurons individually, then we multiplied the computed weights $W_{n}$ to the loss function and generate the mean loss L. The weight values were computed in such a way that this technique will avoid the overfitting to majority classes by decreasing the loss value and avoid the underfitting to minority classes by increasing the loss value. The weight values were calculated as follows:

$$W_{n}=\left(T+\left(K*S_{V}\right)/\left(N*K\right)\right)$$

T = total number of data sample.

$W_{n}$ = weight for current category.

K = number of sample available for current class.

N = total number of classes.

$S_{V}$ = scale value (1, 2, 3, 4, 5, … )

$S_{V}$, a scale value hyperparameter generated promising results with $S_{V}$ = 3. This parameter decides the amount of overfitting. We conducted several experiments to calculate the $S_{V}$ and found that $S_{V}$ = 3 worked better for our use case. We also assume that the right value of $S_{V}$ depends on the overall size of the training data well as the number of instances associated with each label.

### Transfer Learning

In this technique, we have worked with pre-trained models built on a large dataset and the same models were reused to train the task-specific classification. There are two steps in the training process i.e., pre-training and fine-tuning. In case of the BERT classifier, the model was initialized with pre-trained BERT Base-Uncase trained on English Wikipedia and book corpus embeddings. In case of the BioBERT classifier, the model was initialized with pre-trained BioBERT-Base-v1.1 trained on PubMed one million articles embeddings. In case of the XLNet classifier, the model was initialized with pre-trained XLNet-base-case trained on books corpus, English Wikipedia, Giga5, Clue Web 2012-B, and Common Crawl datasets. The important parameter settings used for the fine-tuning are as follows.

training batch size = 32

evaluation batch size = 8

maximum sequence length = 60

number of epochs = 5.

Neural network classifier layers were initialized with Xavier initialization (Glorot and Bengio, 2010) hyper-parameters such as the learning rate. Scheduling for pre-training the multi-class classifier was the same as those for pre-training BERT. We used a single NVIDIA V100 GPU for training and evaluation. During the training phase, we froze the BERT layer and updated only the classification layer, but the results were not optimal. Training the network by updating all the parameters showed good performance but the model converged quickly. Fine-tuning models executed in less than 30 min.

Performances of BERT and BioBERT converged after three epochs whereas XLNet converged after five epochs. In general, the models began to perform well on the training set already after two epochs. Since the data is highly imbalanced, if the cost-sensitive mechanism was not used after the first epoch, it was observed that the performance metrics for some labels with less training data were nearly zero. To achieve good performance for minority classes, we observed the need for training more epochs, but this could eventually lead to overtraining on majority classes. Therefore, the cost-sensitive training becomes very useful in the case of highly imbalanced datasets.

## Results

Table 3 shows the performance of classification with logistic regression trained using different features. The performance metrics used for evaluation were precision, recall, and F1-score. Logistic regression trained using TF-IDF features with imbalanced data indicated low recall. This is because training over minority labels did not converge after few epochs. In the case of logistic regression using USE features and balanced data, the recall was still low. Both linear SVMs and logistic regression trained using TF-IDF features with data balancing techniques performed well and converged faster. The performance of classification on individual labels are provided in Supplementary Table 2.

**TABLE 3:** Results of classification with logistic regression and SVMs using different features evaluated using 10-fold cross validation of the training data with heuristic labels.

Approach	Precision	Recall	F1-score
Logistic regression (imbalanced labels) + TF-IDF	1.00	0.56	0.68
Logistic regression + USE (balanced labels)	0.83	0.51	0.59
Logistic regression (balanced labels) + TF-IDF	0.87	0.90	0.88
Linear SVMs + TF-IDF (balanced labels)	0.9	0.91	0.91

Table 4 shows the results of training and evaluation using different deep learning models. Compared to the results shown in Table 3, deep learning models performed better than classical machine learning classifiers. BioBERT classifier outperformed XLNet and BERT achieving an overall F1-score of 0.95. During the pre-training phase, we observed that in comparison to other models which are trained on open domain data, BioBERT was able to capture the domain knowledge well as it is trained on the PubMed biomedical corpus which has the closest domain to our working corpus. Also, deep learning models are known to handle noisy data and generalize it well. We observed that the deep learning model trained end-to-end performed better when compared to training classifiers using pre-trained embeddings. This holds true for both classical machine learning as well as the deep learning classifiers. The model with BioBERT classifier applied on the leave-out validation set evaluated manually by experts delivered F1-score of 0.83.

**TABLE 4:** Results of classification with transformer models evaluated using 10-fold cross validation of the training data with heuristic labels.

Approach	Precision	Recall	F1-score
BERT classifier	0.97	0.87	0.91
BioBERT Classifier	0.94	0.96	0.95
XLNet Classifier	0.89	0.94	0.91

For the sake of real-world evaluation, a corpus of additional 1978 sentences from inquiries which does not overlap with training or leave-out validation datasets was gathered. They were labeled heuristically as well as using custom trained BioBERT classifier. The scope of real-world evaluation was not to quantitively benchmark heuristics and ML classifier. But the idea was to release the results to multiple experts and collect their qualitative feedback to mitigate any human bias during the leave-out validation process. Four experts were involved in the real-world evaluation process. Since the expert review process is time-consuming and expensive, non-overlapping sentences belonging to specific therapeutic areas were assigned to individual experts. This supported therapy area-specific evaluation, but no inter-reviewer scores could be computed. The results of the qualitative evaluation were subject to expert acceptance of the quality of automatic labeling. Low labeling quality would indeed trigger additional training cycles as well as provide a signal to improve the leave-out dataset validation criteria. Therefore, the real-world evaluation ceases the experimental model development and indicates if the trained model can be used for labeling the bulk data or near real-time predictions.

Sentences where heuristic labels and BioBERT classifier-based labels differ or overlap were manually checked to perform a comparative qualitative assessment. Manual evaluation through spot checks by subject matter experts revealed a relatively better performance of the custom BioBERT model indicating its discriminative power and contextual sensitivity which could not be achieved with heuristics and expert-defined rules alone. Some examples of comparative assessment of heuristic labeling and weakly supervised classification are presented in Table 5.

**TABLE 5:** Examples of types of inquiries as well as the outcome of both heuristics and ML approaches have been presented. Due to data sensitivity, product names have been omitted and original inquiries have been slightly manipulated.

Subcategory type	Sample inquiry	Heuristic approach	Weakly supervised approach
Real world evidence	A customer requested information about clinical trial study data for a patient population of women having a pre-existing condition	For this type of inquiry, the system falsely categorized the inquiry as specific clinical study or trial result	For this type of inquiry, the system was able to predict the accurate category even when the information was not explicitly mentioned in the text
Efficacy	An HCP (healthcare professional) requested information if a patient can be treated with one of our products after undergoing a treatment regimen with other product	In this example, the system was able to correctly identify the category as efficacy since our ruleset was able to capture such patterns of expressions in the data	The system was not able to categorize this inquiry probably due to a smaller number of training examples for the category efficacy
Publication request	A customer requested for scientific references in terms of publication or poster explaining efficacy of one of our products	For this type of inquiries where explicit request for a scientific resource was made, both heuristics and machine learning algorithms were able to categorize the inquiries accurately	

## Conclusion

This article provides an overview of the application of weakly supervised learning for the categorization of medical inquiries. A heuristic approach combined with a proprietary knowledgebase and expert-defined rules were applied to label the data following a two-level categorization hierarchy. Classical machine learning algorithms as well as deep learning algorithms were trained and evaluated using TF-IDF features, pre-trained embeddings, and custom embeddings. The deep learning approach leveraging end-to-end fine-tuned embeddings, dataset balancing, and model training delivered good results. These trained models learned to generalize classification tasks well. They were also able to assign better subcategories that were more relevant in comparison to the heuristics approach. Also, these models were able to assign subcategories where the heuristic-based system failed to assign (i.e., *General or Other* categories). Based on the manual investigation of results of weakly supervised labeling, experts examined that machine learning-driven categorization was able to assign higher quality labels which could potentially have been missed with purely heuristic approach. For example, sentences with expressions like *expensive*, *high price*, *pricy*, and *spend more* were well captured by the machine learning approach and assigned to a uniform label which indeed would have been missed unless explicitly hardcoded within rule set for heuristics. However, the authors highlight the limitation that crafted heuristics and dependent knowledgebases are specific to the dataset used for this research and they cannot be easily generalized to another domain or dataset.

We also propose a methodology to handle the data imbalance problem by using the cost-sensitive loss function which performed well. This technique not only improves the overall classification performance but also overcomes the overfitting to majority labels.

The application of machine learning methods to inquiry category classification is hampered by the necessity of domain knowledge and human efforts to create a large amount of labeled training data which is an expensive operation. Publicly available labeled data sets are not applicable here because the data is domain specific. The proposed solution could solve this problem through weak supervision, automated labeling, and training machine learning algorithms in a supervised manner. As a part of the current roadmap, the solution is planned to be deployed in production setup for business operational use. After successful operationalization, the solution will be continuously measured against the business key performance indicators (KPIs) for business benefits, and effort savings. Additional measures need to be planned to ensure that the risk of model drifts can be systematically traced, and improved models can be quickly redeployed.

## Future Scope

We foresee opportunities for future enhancements both from the scientific methodology as well as business outreach points of view. As a scientific future work for the current solution, we propose that the heuristic rules can be further improved based on the observations from the misclassification as well as machine-generated results. This could improve the labeled data quality for training. Synthetic inquires can also be generated to handle data imbalance, hence more training data for the minority classes. Regular user feedback can be incorporated, and principles of active learning and reinforcement learning can be investigated to check if the classification performance can be eventually improved.

From a business driver point of view, the current solution can potentially be improvised and customized for additional business areas within our organization where similar challenges are observed. Here are few such examples where improvised version of this solution can be extended but not limited to 1) adding potentially new data sources like Customer Relationship Management (CRM) systems and social media data to improve the scope of the analytics and insights 2) from near real-time analytics to real-time recommendation system for contact center employees to ensure consistent and accurate feedback to customers. With a data-driven approach, we foresee potentially new business areas where the learnings can be cross-pollinated to enhance the customer experience for our products. Ideally, the goal is to provide a decision support system that can span beyond the Medical Information organization and potentially adding value to organizational cross-sectors such as research, development, marketing, sales, manufacturing, supply chain, and post-market intelligence.

## Data Availability

The data analyzed in this study is subject to the following licenses/restrictions: proprietary data. Requests to access these datasets should be directed to harsha.gurulingappa@merckgroup.com.
