# Supplementary material for: Weakly Supervised Learning for Categorization of Medical Inquiries for Customer Service Effectiveness
Source: Front Res Metr Anal. 2021 Aug 2;6:683400. doi: 10.3389/frma.2021.683400 (PMC8366288; doi:10.3389/frma.2021.683400)
Supplement: Supplementary file 1 [file Table1.DOCX]

Supplementary Material

| **Label** | **Subcategory** | **#Sentences with heuristic label assigned** | **Labeling Criteria** |
| --- | --- | --- | --- |
| 0 | **Dosage and Administration** | 13634 | - Recommended dosage and modifications (e.g., for a specific patient sub-population such as renal impairment, hepatic impairment, pediatrics, etc.) - Instructions on how to use or administer a product (e.g., before meal, with meal, etc.) - Information regarding calculations needed to determine dosage for specific patient populations. - Alternative routes of administration, dosing, frequency (e.g., opening capsules, splitting tablets, crushing tablets, every other day dosing, higher/lower than recommended dosage, etc.) |
| 1 | **Formulations (pharmaceutics/ingredients)** | 2927 | - Ingredient inquiries (e.g., Does this drug contain gluten? What is the sodium content in this tablet?) - How supplied (color, size, quantity, shape of medication, NDC number) |
| 2 | **Storage, Stability and Handling** | 9979 | - Temperature excursions - Repackaging medication - Expiration dates - Shelf life - Beyond use date - Disposal - MSDS requests |
| 3 | **Switching/Transition** | 1633 | - Inquiries regarding the switching from one drug to another drug. - Dose conversion (what is the equivalent dosage when switching from Drug A to Drug B?) - Inquiries regarding dose titration or dose tapering |
| 4 | **Pharmacology and Pharmacokinetics** | 15362 | - Pharmacokinetics, pharmacodynamics, mechanism of action, etc. - Process to understand how the specific indication in patients is affected after administration of drugs. - Absorption, Distribution, Metabolism, Elimination (ADME) |
| 5 | **Drug Interactions** | 374 | - Interactions between one drug and another treatment or lifestyle factor (e.g., Drug-Drug Interactions, Drug-Food Interactions, Drug-Smoking interaction) |
| 6 | **Efficacy Data** | 9935 | - Request for efficacy information for an approved indication or unapproved use (e.g., potential future indication) |
| 7 | **Specific Clinical Study or Trial Results** | 7330 | - Specific queries on efficacy and safety in comparison to another drug or placebo in a named/specific study or trial - Information request for results of a specific study or trial - Overall results and outcomes for a study or trial with no specific mention of the product |
| 8 | **Comorbidities and Special Patient Populations** | 480 | - Reference to usage of drug in any sub-group of populations for a specific condition – post amputation, renal impairment, hepatic impairment, reduced ejection fraction, short bowels, Hispanic patients, etc. - Results in the context of a specific sub-group of patient population enrolled for a specific study. |
| 9 | **Comparison to other Drugs or Products** | 425 | - Specific queries on efficacy or safety of a drug in comparison to another drug (e.g., specific head-to-head study or trial, rates of an adverse event versus another drug in class, etc.) |
| 10 | **Combination Therapy** | 981 | - Specific queries on use of specific drug in combination with another drug for a same or different indication area. |
| 11 | **Real World Evidence** | 219 | - Corresponds to the clinical evidence regarding the usage and potential benefits or risks of drugs derived from analysis of Real-World Data. - Request for real world evidence (RWE), clinical practice data |
| 12 | **Safety Data** | 21290 | - Request for Information on specific adverse event related to a drug - Information on study results on specific side effect for a drug – incidence of skin rash with specific products etc. - Safety information needed in a specific sub-group of population enrolled in the study. - Mechanism of a particular Adverse Event (theoretical or known) |
| 13 | **General Disease State Information** | 4566 | - Information requested not specific to a product but for the therapeutic area. It could be for the underlying disease area or areas supporting the therapy. - No mention of the product but there could be a reference made to the product class. |
| 14 | **Clinical Practice Guidelines** | 876 | - Can include diagnosis or treatment guideline requests, including inquiries regarding the place of a specific drug in the treatment guidelines |
| 15 | **Publication request** | 4811 | - Explicit request for a scientific resource (reprint/copy of article, manuscript, abstract, poster, slide decks, pdf, etc.) - Mention of an HPRC/PubMed identifiers (e.g., SC-US-12345 or PC-US-12345) - Request for appendix/supplement of studies. |
| 16 | **Meeting request** | 2517 | - In addition to requesting information an explicit request for MSL or AMA, medical team member, face-to face meeting with the HCP |
| 17 | **Patient support** | 2627 | - HCPs asking for patient assistance programs, coupons, insurance queries, prior authorizations, patient support hubs, starter kit, etc. - Any educational materials for the patients (including other languages) - Package Inserts/Medication guide in other languages (e.g., Spanish, Korean, Chinese, etc.) |
| 18 | **Formulary** | 298 | - Documents regarding a specific Academy of Managed Care Pharmacy (AMCP) dossier, e-dossier, Health Economics and Outcomes Research (HEOR), health economic data, cost effectiveness model, budget impact model, Pharmacy and Therapeutics (P&T) for certain drugs. |
| 19 | **Access and Availability** | 9271 | - Backorder inquiries - When will product be available? - When was the drug launched? - Is a generic available? - Where to fill prescriptions or become an authorized distributor for Specialty Medications |
| 20 | **Comments and Suggestions** | 381 | - Extraneous neutral comments - Thoughts on changes in packaging or look of drug |
| 21 | **Complaints** | 567 | - Negative feedback (product, advertisement or website feedback) - Cost complaints |
| 22 | **Compliments** | 131 | - Positive feedback (product, advertisement or website feedback) - Congratulating the company for developing the product and making it available for patients |

Supplementary Table 1: Definitions for subcategories and counts of sentences tagged with subcategory labels using heuristics.

|  | XLNet | | | BioBERT | | | BERT | | |
| --- | --- | --- | --- | --- | --- | --- | --- | --- | --- |
| **Category** | **Precision** | **Recall** | **F1-score** | **Precision** | **Recall** | **F1-score** | **Precision** | **Recall** | **F1-score** |
| 0 | 0.99 | 0.97 | 0.98 | 1 | 0.97 | 0.98 | 0.97 | 0.91 | 0.94 |
| 1 | 0.94 | 0.99 | 0.96 | 0.99 | 0.99 | 0.99 | 1 | 0.99 | 0.99 |
| 2 | 0.98 | 0.96 | 0.97 | 1 | 0.98 | 0.99 | 1 | 0.98 | 0.99 |
| 3 | 0.69 | 0.94 | 0.79 | 0.89 | 0.99 | 0.94 | 1 | 0.98 | 0.99 |
| 4 | 0.84 | 0.97 | 0.9 | 0.96 | 0.96 | 0.96 | 0.98 | 0.98 | 0.98 |
| 5 | 0.96 | 0.96 | 0.96 | 0.79 | 0.99 | 0.88 | 0.84 | 0.96 | 0.9 |
| 6 | 0.85 | 0.98 | 0.91 | 0.97 | 0.96 | 0.96 | 0.98 | 0.95 | 0.96 |
| 7 | 0.69 | 0.95 | 0.8 | 0.73 | 0.93 | 0.82 | 1 | 0.93 | 0.96 |
| 8 | 0.68 | 0.69 | 0.68 | 0.83 | 0.83 | 0.83 | 1 | 0.78 | 0.88 |
| 9 | 0.73 | 0.98 | 0.84 | 0.98 | 1 | 0.99 | 0.98 | 1 | 0.99 |
| 10 | 1 | 0.97 | 0.99 | 0.99 | 0.99 | 0.99 | 0.98 | 0.81 | 0.89 |
| 11 | 0.99 | 0.96 | 0.97 | 0.99 | 0.96 | 0.98 | 0.98 | 0.92 | 0.95 |
| 12 | 0.97 | 0.96 | 0.96 | 0.99 | 0.97 | 0.98 | 0.99 | 0.94 | 0.96 |
| 13 | 0.99 | 0.96 | 0.98 | 1 | 0.99 | 0.99 | 1 | 0.96 | 0.98 |
| 14 | 0.91 | 0.98 | 0.94 | 0.99 | 0.99 | 0.99 | 0.98 | 0.97 | 0.98 |
| 15 | 0.97 | 0.95 | 0.96 | 1 | 0.98 | 0.99 | 1 | 0.93 | 0.96 |
| 16 | 0.97 | 0.79 | 0.87 | 0.97 | 0.77 | 0.86 | 0.89 | 0.2 | 0.33 |
| 17 | 0.98 | 0.97 | 0.98 | 1 | 0.99 | 1 | 1 | 0.93 | 0.96 |
| 18 | 0.43 | 0.82 | 0.56 | 0.6 | 0.86 | 0.7 | 0.93 | 0.76 | 0.84 |
| 19 | 0.99 | 0.95 | 0.97 | 1 | 0.95 | 0.97 | 0.76 | 0.43 | 0.55 |
| 20 | 0.99 | 0.96 | 0.97 | 1 | 0.99 | 0.99 | 1 | 0.93 | 0.96 |
| 21 | 0.99 | 0.98 | 0.98 | 1 | 0.99 | 0.99 | 0.97 | 0.91 | 0.94 |
| 22 | 0.95 | 0.99 | 0.97 | 0.98 | 0.99 | 0.98 | 0.99 | 0.97 | 0.98 |
| **Macro Avg** | **0.89** | **0.94** | **0.91** | **0.94** | **0.96** | **0.95** | **0.97** | **0.87** | **0.91** |

Supplementary Table 2: Performance of classification on individual labels using different approaches evaluated through 10-fold cross validation.
